# Design and preclinical evaluation of a universal SARS-CoV-2 mRNA vaccine

**DOI:** 10.3389/fimmu.2023.1126392

**Published:** 2023-03-23

**Authors:** Jane Qin, Ju Hyeong Jeon, Jiangsheng Xu, Laura Katherine Langston, Ramesh Marasini, Stephanie Mou, Brian Montoya, Carolina R. Melo-Silva, Hyo Jin Jeon, Tianyi Zhu, Luis J. Sigal, Renhuan Xu, Huabin Zhu

**Affiliations:** ^1^ Research and Development Department, Advanced RNA Vaccine Technologies, Inc., North Bethesda, MD, United States; ^2^ Department of Microbiology and Immunology, Thomas Jefferson University, Philadelphia, PA, United States; ^3^ Department of Biology, University of Maryland, College Park, MD, United States; ^4^ Greenbrier High School, Evans, GA, United States

**Keywords:** SARS-CoV-2, mRNA vaccine, neutralizing antibody, T cell response, multiple T cell epitopes (MTEs)

## Abstract

Because of the rapid mutations of severe acute respiratory syndrome coronavirus 2 (SARS-CoV-2), an effective vaccine against SARS-CoV-2 variants is needed to prevent coronavirus disease 2019 (COVID-19). T cells, in addition to neutralizing antibodies, are an important component of naturally acquired protective immunity, and a number of studies have shown that T cells induced by natural infection or vaccination contribute significantly to protection against several viral infections including SARS-CoV-2. However, it has never been tested whether a T cell-inducing vaccine can provide significant protection against SARS-CoV-2 infection in the absence of preexisting antibodies. In this study, we designed and evaluated lipid nanoparticle (LNP) formulated mRNA vaccines that induce only T cell responses or both T cell and neutralizing antibody responses by using two mRNAs. One mRNA encodes SARS-CoV-2 Omicron Spike protein in prefusion conformation for induction of neutralizing antibodies. The other mRNA encodes over one hundred T cell epitopes (multi-T cell epitope or MTE) derived from non-Spike but conserved regions of the SARS-CoV-2. We show immunization with MTE mRNA alone protected mice from lethal challenge with the SARS-CoV-2 Delta variant or a mouse-adapted virus MA30. Immunization with both mRNAs induced the best protection with the lowest viral titer in the lung. These results demonstrate that induction of T cell responses, in the absence of preexisting antibodies, is sufficient to confer protection against severe disease, and that a vaccine containing mRNAs encoding both the Spike and MTE could be further developed as a universal SARS-CoV-2 vaccine.

## Introduction

The emergence of coronavirus disease 2019 (COVID-19) rapidly induced a global public health emergency. According to the World Health Organization, as of October 4, 2022, there have been more than 615 million confirmed cases worldwide and over 6 million confirmed deaths (1). In addition, almost one billion people in lower-income countries have not had access to life-saving vaccines and remain unvaccinated (2). COVID-19 continues to spread rapidly and evolve as the virus, severe acute respiratory syndrome coronavirus 2 (SARS-CoV-2), the causative agent of COVID-19, changes over time. These changes may affect the pathological properties of the virus, such as its rate of infection and disease severity, as well as the performance of vaccines, therapeutics, diagnostic tools, or other public health and social measures (3). One of the challenges that threaten the performance and efficiency of vaccines is the emergence of novel viral variants, which are more contagious (4) and have the ability to infect a broader range of host species (5). Currently, there are two variants of concern (VOC), the Delta variant (6) and the Omicron variant, which includes BA.1, BA.2, BA.3, BA.4, BA.5, and descending lineages (7). A continuing concern is the ability of SARS-CoV-2 variants to emerge repeatedly with the ability to escape vaccine immunity (8, 9). Variant-updated vaccines and multiple rounds of immunization are essential to control viral spread. Many countries struggle with repeated waves of infection and do not have sufficiently effective vaccines against newly circulating viral variants. Therefore, further COVID-19 vaccine development is necessary.

The most common strategy in current vaccine platforms is the use of the Spike protein of the SARS-CoV-2 virus as the only antigen. These vaccines aim to induce anti-Spike neutralizing antibodies that specifically bind to the receptor binding domain (RBD) to block the entry of the virus into the host cell (10, 11). This strategy is effective with RNA vaccines showing up to 95% efficacy (10, 12). However, newly emerging VOCs threaten the efficacy of these vaccines because of mutations in Spike protein, allowing the virus to evade antibody-based immunity (13, 14). As a result, new strategies are needed to combat new VOCs.

Potent T cell responses are imperative to adaptive immunity (15). Moreover, conserved T cell responses can be particularly important when new viral variants evade the neutralizing antibodies (15). A number of clinical studies have shown that T cells induced by natural infection with SARS-CoV-2 or vaccination contribute significantly to the protective effect of COVID-19 (16, 17). Thus, if T cell epitopes are derived from conserved regions of the virus, T cell-inducing vaccines have the potential to be an alternative strategy for the development of a universal COVID-19 vaccine.

To address whether a broad COVID-19 vaccine could be achieved by unitizing T cell immunity that recognizes the conserved region of SARS-CoV-2, we designed a universal COVID-19 vaccine which is a lipid nanoparticle (LNP) formulated mRNA vaccine containing two mRNAs. One mRNA encodes SARS-CoV-2 Omicron S protein in prefusion confirmation for induction of neutralizing antibodies. The other mRNA encodes over one hundred T cell epitopes derived from non-Spike conserved regions of SARS-CoV-2. These multi-T cell epitopes (MTE) are conserved across all known SARS-CoV-2 variants, as well as other members of the coronavirus family. Our results show that immunization with MTE alone is sufficient to protect mice from lethal challenge in two mouse models. Immunization with both mRNAs induced the best protection with the lowest viral titer in the lung. Notably, these protections were achieved using 0.1 μg mRNA.

## Materials and methods

### DVS, OVS, and MTE mRNA and LNP formulation

**Figure 1:**
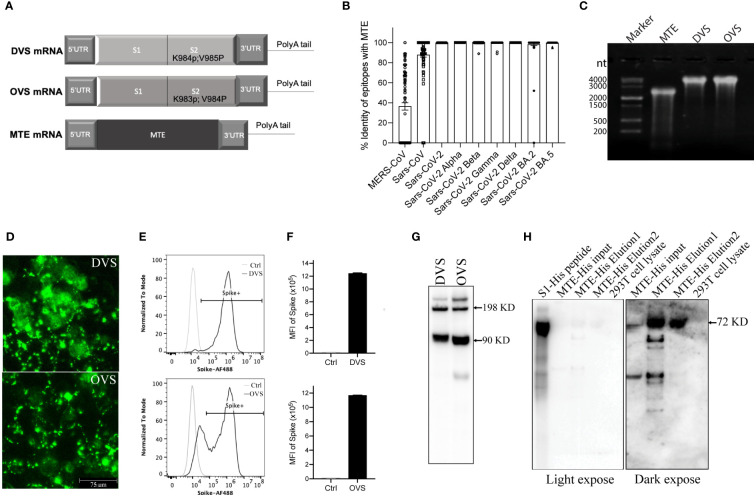
Design and validation *in vitro* of mRNA vaccine. **(A)** Schematic of SARS-CoV-2 Spike protein Delta (DVS) and Omicron (OVS) variants, as well as MTE mRNA. **(B)** Identity of epitopes of MTE with MERS-CoV, SARS-CoV and SARS-CoV-2 BA.2 variant. **(C)** MTE, DVS, and OVS mRNAs were synthesized with T7 RNA polymerase *in vitro* transcription and run on 0.8% MOPS agarose gel. **(D–G)** DVS, and OVS mRNA were synthesized with *in vitro* T7 transcription reactions and transfected into 293T cells with mRNA transfection kit. The expression level of DVS and OVS in 293T cells was detected by fluorescence microscopy **(D)** flow cytometry **(E, F)** and western blot **(G)**. **(H)** MTE-His mRNA was transfected into 293T cells and precipitated with His tag column. Eluted samples were detected by western blot.

Spike sequences in this study were derived from SARS-CoV-2 Wuhan strain, accession ID NC_045512 and modified for Delta variant Spike (DVS) and Omicron variant Spike (OVS) according to the Delta variant and Omicron variant mutation on the CDC website. Sequences of SARS-CoV-2 DVS, OVS, and MTE mRNA were codon optimized and inserted into PUC57, which contains a T7 promoter, 5’-UTR, 3’-UTR, and polyA tail (**Figure 1A**). MTE mRNA consists of hundreds of T cell epitopes derived from all genes except for the S gene, including structural protein E, M and N and other open reading frames encoding nonstructural proteins. T cell epitopes are based on published sequences (18–20) and predicted sequences using IEDB web server-based MHC T cell epitope identification tool as described previously (21, 22). Plasmid constructs PUC-DVS, PUC-MTE and PUC-OVS were synthesized by GenScript. DVS, OVS, and MTE mRNA were made with T7 polymerase *in vitro* transcription (IVT) using pseudo-UTP. The capping was done after the completion of IVT, the IVT product was purified first then subjected to capping reaction. We use the dual enzyme capping reaction, Vaccinia Capping System (NEB, Cat # M2080B-1ml) and MTE (NEB, M0266B-1ml).

Lipids used for LNP fabrication were ionizable lipid heptadecan-9-yl 8-[2-hydroxyethyl-(6-oxo-6-undecoxyhexyl)amino]octanoate (SM-102) purchased from Broadpharm (BP-25499). Helper lipid 1,2-distearoyl-sn-glycero-3-phosphocholine (DSPC,850365C-1g), cholesterol (700100P), and 1,2-dimyristoyl-snglycero- 3-phosphoethanolamine-N-[methoxy(polyethylene glycol)-2000] (DMG-PEG-2000, 880151P-1g), purchased from Avanti Polar Lipids. L002 is a candidate of ARV proprietary ionizable lipid.

The formulations were prepared by mixing lipids in an organic phase with an aqueous phase containing mRNA using a Nanoassemblr^®^ Ignite microfluidic device (Precision NanoSystems). The molar percentage ratio for the constituent lipids is 50% for L002 or SM-102, 10% DSPC, 38.5% cholesterol, and 1.5% DMG-PEG. At a flow ratio of 1:3 organic: aqueous phases, the solutions were combined in the microfluidic device. The total combined flow rate was 12 mL/min per microfluidics chip. The LNP-mRNA mixture was dialyzed and concentrated by centrifugation.

For mRNA quantification post formulation, we applied RiboGreen assay. We followed the manufacturer’s protocol (Thermo Fishers: Quant-iT™ RiboGreen™ RNA Reagent and Kit, Cat# R11490, R11491, T11493. Pub. No. MAN0002073). For mRNA quality control after formulation, we isolated mRNA from the LNPs and performed agarose gel electrophoresis.

### Expression of DVS, OVS, and MTE mRNA *in vitro*

DVS, OVS, and MTE mRNA were transfected into 293T cells with MessengerMax (Invitrogen). After 48h, we evaluated the expression of the three mRNAs with flow cytometry (FC), immunofluorescence (IF), and western blot (WB). For IF, cells were incubated with rabbit anti-s1 antibody (Sino Biological) and AF488 conjugated anti-Rabbit secondary antibody (Abcam), and images were taken with the machine. For FC, cells were trypsinized and incubated with rabbit anti-s1antibody (Sino Biological) and AF488 conjugated anti-Rabbit secondary antibody (Abcam). Data was acquired with C6 (BD Biosciences) and analyzed with FlowJo (BD Biosciences). For WB, cells were harvested and denatured in lysis buffer. Samples were loaded and run in 4-12% gradient SDS-PAGE gel and transferred to the PVDF membrane. PVDF membrane was incubated with mouse anti-s2 monoclonal antibody (Thermofisher, Cat# MA5-35946) and HRP conjugated anti-mouse secondary antibody (Invitrogen, Cat#62-6520). Anti-beta-Actin HRP Antibody for protein loading control was purchased from Santa Cruz Biotechnology (sc-47778 HRP).

### Mice and peptides

Six to eight-week-old female BALB/c mice were bred and maintained at an animal facility in Noble Life Sciences (Woodbine, MD). For immunogenicity studies, mice were immunized intramuscularly with formulated mRNA or PBS as indicated at Day 0 (prime) and Day21 (boost). Serum was collected after two weeks of prime and boost. Mice were euthanized at Day35, and spleens were collected for analysis of T cell immunity.

In the challenge study with SARS-CoV-2 Delta variant, K18-hACE2 transgenic mice were maintained at Bioqual Inc. (Rockville, MD) and immunized intramuscularly with formulated mRNA or PBS at Day 0 and 21. Serum was collected at Day 28 for antibody testing. At Day 35, mice were challenged intranasally with SARS-CoV-2 Delta variant at a dose of 5 × 10^3^ the median tissue culture infectious dose (TCID_50)_ SARS-CoV-2 B.1.617.2 (Delta) (BEI Resources SARS-CoV-2, isolate hCoV-19/USA/MDHP05647/2021, NR-55674). Mouse body weight and survival were recorded daily. At 4 days post of infection (DPI), 5 mice of each group were euthanized, and mouse tissues were collected and stored in Trizol or formalin.

In the challenge study with mouse adapted SARS-CoV-2 MA30, BALB/c mice were maintained at animal facility of Thomas Jefferson University (Philadelphia, PA) and immunized intramuscularly with formulated mRNA or PBS at Day 0 and 21. Serum was collected at Day 28 for antibody testing. At Day 35, mice were challenged intranasally with mouse adapted SARS-CoV-2 MA30 at a dose of 5 x 10^3^ PFU in volume of 50 µL. Mouse body weight and survival were recorded post challenge. At four DPI, 5 mice of each group were euthanized, and mouse tissues were collected and stored in Trizol.

MTE overlapping peptides were synthesized by GenScript (Piscataway, NJ). Spike S1 overlapping peptides were purchased for JPT (Berlin, Germany).

### Evaluation of antigen-specific T cell response by ELISPOT

Splenocytes from vaccinated mice were evaluated for antigen-specific IFN𝝲 by Enzyme-linked immunospot (ELISPOT). ELISPOT assays were performed as per ARV SOP. Briefly, a 96-well ELISPOT plate (Millipore, Cat#MSIPS4510) was coated with 10 µg/mL IFN𝝲 antibody (Biolegend, Cat# 517902, clone AN18) at 4°C overnight. Splenocytes were plated at 3x10^5^ cells/well and co-cultured with either 0.5 µg/mL Spike peptides (JPT, Cat#PM-WCPV-S-1), 2 µg/mL human MTE overlapping peptides (synthesized by Genscript), concanavalin A (0.125 ug/mL) (Sigma, Cat#C5275-5MG), or medium alone in a total volume of 200 µL/well T cell media for 48h at 37°C in 5% CO2. The plates were incubated with detection antibodies, Biotin-IFN𝝲 (Biolegend, Cat# 505714, clone R46A2) and Streptavidin-HRP (Biolegend, Cat# 405210) at RT for 1-2 hours, respectively. The plates were developed with 50 µL/well AEC development solution (BDbiosciences, Cat#551015) for up to 30 min. Color development was stopped by washing under running tap water. After air-dried, colored spots were counted using an AID ELISPOT High-Resolution Reader System and AID ELISPOT Software version 3.5 (Autoimmun Diagnostika GmbH).

### ELISA

The murine antibody response to the Spike was assessed by indirect ELISA. ELISA plates (Nunc MaxiSorp, Thermofisher, Cat#44-2404-21) were coated with 1 mg/mL recombinant Spike protein (Sino Biological Inc, Cat#40591-V08H) overnight and then blocked with 2% BSA in PBS. Serum samples were diluted by 200x, followed by a 1:5 serial dilution for up to 8 wells with 0.2% BSA in PBS. Samples were detected with 1:2000 goat anti-*mouse IgG-HRP* (Southern Biotech, Cat# 1031-05). The reaction was developed with TMB Substrate (Sigma, Cat#T0440-1001) and stopped with TMB Stop Solution (Invitrogen, Cat#SS04). Plates were read at OD450 using an Epoch ELISA reader (BioTek, Winooski, VT).

### Lentivirus-based pseudovirus neutralization assay

The SARS-CoV-2 pseudoviruses, including the Delta variant and Omicron variant expressing a luciferase reporter gene, were purchased from Codex BioSolutions (Rockville, MD). HEK293T-hACE2 cells were seeded in 384-well tissue culture plates at a density of 7.5 × 10^3^ cells per well overnight. Two-fold serial dilutions of heat-inactivated serum samples were prepared (17.5 µL) and mixed with 7.5 µL of pseudovirus. The mixture was incubated at 37°C for 1h before adding to HEK293T-hACE2 cells. After 48h, cells were lysed in Steady-Glo Luciferase Assay (Promega) according to the manufacturer’s instructions. SARS-CoV-2 neutralization titres were defined as the sample dilution at which a 50% reduction in relative light units (IC_50_) was observed relative to the average of the virus control wells.

### Immunohistochemistry

Mouse lung tissues were fixed in 10% neutral-buffered formalin and embedded in paraffin. Sections of tissue with 5 µm in thickness were affixed to slides. Slides were stained with hematoxylin and eosin or Nucleocapsid antibody of SARS-CoV-2 (CST, Cat# 26369s) according to the standard program.

### Statistical analysis

Statistical significance was calculated using Student’s *t*-test with two-tailed analysis. A p-value less than 0,05 (p < 0.05) was considered statistically significant.

## Results

### mRNA design and expression *in vitro*

DVS mRNA encodes the full-length Spike protein from the SARS-CoV-2 Delta variant, in which we replaced amino acid lysine at 984 and valine at 985 with proline (SP2) for prefusion conformation (23) (**Figure 1A**). OVS mRNA encodes the full-length Spike protein from the SARS-CoV-2 Omicron variant, in which we replaced amino acid lysine at 983 and valine at 984 with proline (SP2) for prefusion conformation (**Figure 1A**). T cell epitopes are ~8-11 or ~13-25 amino acid long peptides respectively presented to CD8^+^ and CD4^+^ T-cells by MHC class I (MHC I) or MHC class II (MHC II) molecules. MTE mRNA encodes approximately one hundred T cell epitopes including both MHC I and MHC II from conserved sequences of SARS-CoV-2 Wuhan strain (**Figure 1A**). Some T cell epitopes strongly cross-react with mouse T cells, which has been experimentally validated (data not shown). Thus, MTE vaccine is able to induce T-cell immune responses in mice. The sequence of each MTE epitope was compared and scored by percentage identity with those of Coronavirus including MERS-CoV, SARS-CoV and SARS-CoV-2 variants. T cell epitopes in MTE have 36.4% identity with MERS-CoV, 87.9% identity with SARS-CoV, 98.3% identity with SARS-CoV-2 omicron BA.2 variant, and more than 99.8% identity with SARS-CoV-2 Alpha variant, Beta variant, Gamma variant, Delta variant and Omicron BA.5 variant (**Figure 1B**). Further analysis found that there were over twenty epitopes in MERS-CoV with more than 70% identity (**Supplementary Table 1**). These data indicated sequences of T cell epitopes in MTE were highly conserved in SARS-CoV, SARS-CoV-2, as well as SARS-CoV-2 variants.

DVS, OVS, and MTE mRNAs were produced by IVT with T7 polymerase (**Figure 1C**). DVS and OVS mRNA were transfected in 293T cells and their expression was detected with the anti-Spike antibody. Immunofluorescent staining showed membrane localization of DVS and OVS protein (**Figure 1D**). Flow cytometry confirmed cell surface localization and high level of protein expression of DVS (MFI 1.24M) and OVS (MFI 1.17M) (**Figures 1E, F**). DVS and OVS proteins were also detected by Western blotting under denaturing conditions with mouse anti-Spike S2 monoclonal antibody. Two major bands were visible, corresponding to 90 KD S2 protein, and 198 KD full length of Spike protein (24) (**Figure 1G**).

Because there is no antibody available for MTE detection, in addition to MTE mRNA, MTE-His mRNA was parallelly constructed to tag MTE with six histidine at the C terminal. After transfection into 293 T cells, cells were lysed and MTE-His fusion protein was concentrated with His tag purification column kit (SigmaAldrich, Cat# H7787), followed by Western blotting under denaturing conditions. An expected size of 72KD major band and smaller molecular weight degraded products were observed in the first and second elution (E1 and E2) (**Figure 1H**), suggesting rapid degradation of MTE fusion proteins.

### Immunogenicity in BALB/c mice

**Figure 2:**
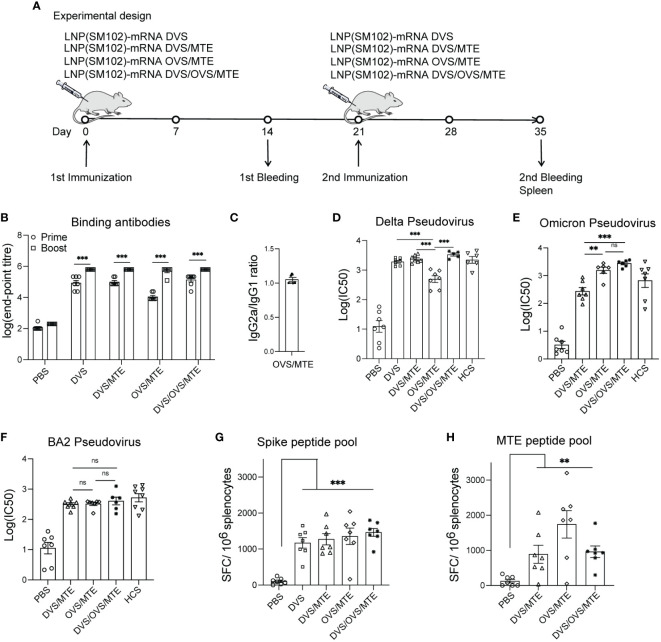
Universal vaccines elicits robust specific immunogenicity in BALB/c mice. **(A)** BALB/c mice were intramuscularly immunized on D0 and D21 with 10 µg of LNP(SM102) formulated mRNA vaccines (DVS or DVS/MTE or OVS/MTE or DVS/OVS/MTE). Mouse sera were collected on D14 (prime) and D35 (boost). Mouse spleens were collected on D35. **(B, C)** Spike-specific IgG **(B)** or subtype IgG1 and IgG2a **(C)** was detected by ELISA from serum samples. D-F, Neutralization assays were performed with different variants of SARS-CoV-2 pseudovirus, Delta variant **(D)**, Omicron variant **(E)**, and BA.2 variant **(F)**. **(G, H)** Splenocytes were isolated from mouse spleen and performed for ELISPOT assay with stimulation of a Spike peptide pool **(G)** or MTE peptide pool **(H)**. *P<0.05, **P<0.01, ***P<0.001; ns, presents not significant.

The strategy of our universal vaccines against SARS-CoV-2 is based on neutralizing antibodies (induced by Spike mRNA) and T cell responses (induced mainly by MTE mRNA). Next, we mixed either OVS mRNA or DVS mRNA with MTE mRNA at a ratio of 1:1 and formulated the RNA into SM102-based LNPs, referred to as LNP(SM102)-mRNA OVS/MTE and LNP(SM102)-mRNA DVS/MTE. We also prepared LNP(SM102)-mRNA DVS and LNP(SM102)-mRNA DVS/OVS/MTE as a control. BALB/c mice were immunized intramuscularly with 10 µg of LNP-mRNA vaccines at Day 0 (prime) and D21 (boost), sera were collected two weeks after prime and boost, and spleens were collected at D35 (**Figure 2A**). First, we measured Spike-specific IgG antibodies from serum after prime and boost by ELISA. The same pattern was observed in the generation of Spike-specific antibodies and there was no significant difference in Spike binding antibody IgG titres between DVS and DVS/MTE. The logarithm of IgG titres of Spike-specific antibodies were 4.93 (DVS) vs. 4.99 (DVS/MTE) after prime and 5.80 (DVS) vs. 5.80 (DVS/MTE) after boost (**Figure 2B**). The logarithm of IgG antibody titres in OVS/MTE (4.04) were slightly lower at D14 than DVS/MTE (5.0) and DVS/OVS/MTE (5.14) but were comparable with the other two groups at D35 (DVS/MTE vs. OVS/MTE vs. DVS/OVS/MTE were 5.8 vs. 5.7 vs. 5.7) (**Figure 2B**). It has been reported that the antigenicity of the Omicron Spike protein is different from previous variants (25). This could explain why the IgG titres were lower at D14 with the OVS/MTE vaccine.

Biases of either Th1 cells or Th2 cells prior to vaccine administration were also reported to be associated with immune disease (26). The percentage of IgG2a/IgG1 determined the Th1/Th2 ratio. The balance of Spike-specific IgG2a/IgG1 in the LNP-mRNA DVS/MTE group was 1.05. No Th1 or Th2 bias was detectable with our LNP-mRNA formulation (**Figure 2C**).

We then evaluated the inhibition of viral entry with mouse serum using Delta pseudovirus, Omicron pseudovirus, and BA.2 pseudovirus in 293T-hACE2 cells. As a control, DVS and DVS/MTE groups were evaluated using Delta pseudovirus. The logarithm of IC_50_ of the neutralizing antibody was comparable in mice immunized with LNP-mRNA DVS (3.28) or LNP-mRNA DVS/MTE (3.38). The titres were also similar to human COVID-19-convalescent serum (HCS) (3.35) (**Figure 2D**). As expected, the neutralizing antibody (the logarithm of IC_50_) to Delta pseudovirus was detectable but lower in the OVS/MTE group (2.69), compared to DVS/MTE group (3.38), DVS/OVS/MTE group (3.53) and HCS (3.35) (**Figure 2D**). Reciprocally, the neutralizing antibody to Omicron pseudovirus were detectable but lower in the DVS/MTE group (2.43), compared to the OVS/MTE group (3.20), DVS/OVS/MTE group (3.44) or HCS (2.82) (**Figure 2E**). The neutralizing antibody to BA.2 pseudovirus was much lower in all groups. The logarithm of IC_50_ were 2.49 in the DVS/MTE, 2.51 in the OVS/MTE, 2.56 in the DVS/OVS/MTE group, and 2.72 in the HCS control (**Figure 2F**). Thus, the neutralizing antibody titres against BA.2 variant were lower than those against Delta and Omicron BA.1 variant, consistent with previous report (27). Therefore, the reduced neutralization of heterogeneous variants suggests the importance of developing an antibody-independent but universal SARS vaccine.

Next, we determined the T cell responses using ELISPOT assay with splenocytes stimulated with a Spike peptide pool or an MTE peptide pool. As a control, DVS and DVS/MTE groups were evaluated following stimulation with a Spike peptide pool. There were 1172 and 1272 spot-forming cells (SFC)/10^6^ splenocytes in LNP-mRNA DVS and LNP-mRNA DVS/MTE immunized BALB/c mice, respectively. The results showed no significant difference in T cell responses between two groups (**Figure 2G**). The numbers of SFC/10^6^ splenocytes in the DVS/MTE, OVS/MTE and DVS/OVS/MTE groups were, respectively, 1272, 1355, 1464 following stimulation with a Spike peptide pool (**Figure 2G**) and 891, 1742, 960 following stimulation with a MTE peptide pool (**Figure 2H**). The results showed that all the groups elicited strong Spike-specific T cell response or MTE-specific T cell response, respectively.

Together, these data indicated that integrating two mRNAs in one LNP formulation has similar immunogenicity to an LNP formulation utilizing one mRNA. In addition, DVS/MTE, OVS/MTE, and DVS/OVS/MTE elicited a robust Spike-specific antibody response and strong Spike and MTE-specific T cell responses. We selected OVS/MTE for further evaluation.

### LNP(L002)-formulated mRNA vaccine elicits robust immune responses at a low dose

**Figure 3:**
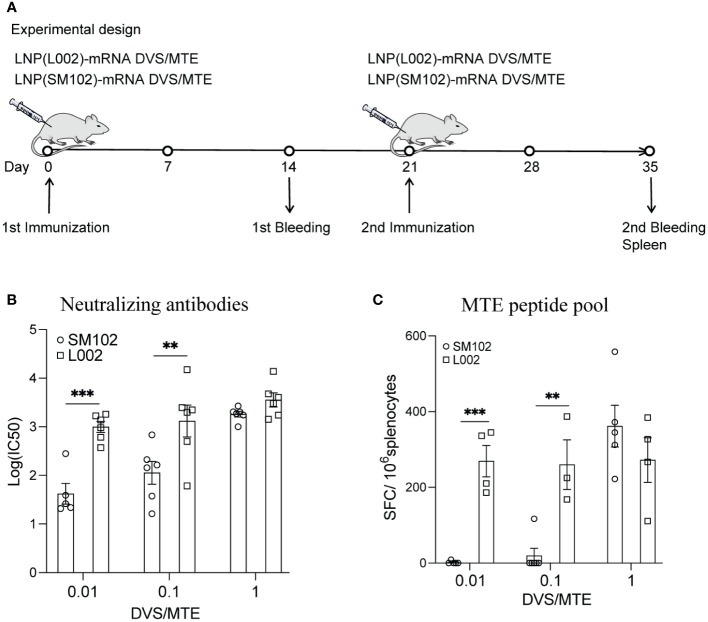
LNP(L002)-mRNA DVS/MTE vaccine elicit more robust specific immunogenicity than LNP(SM102)-mRNA DVS/MTE vaccine. **(A)** BALB/c Mice were immunized with 0.01, 0.1 or 1 µg of either LNP(L002)-mRNA DVS/MTE or LNP(SM102)-mRNA DVS/MTE vaccine on D0 and D21. Mouse sera were collected on D14 (prime) and D35 (boost). Mouse spleens were harvested on D35. **(B)** Serums from D35 were assessed for neutralizing antibodies against SARS-CoV-2 Delta pseudovirus. **(C)** Splenocytes from mouse spleen were isolated and performed for ELISPOT assay with stimulation of an MTE peptide pool. **P<0.01, ***P<0.001.

Besides formulating mRNA into SM102-based LNPs, we also formulated them into a new L002 ionizable lipid-based LNPs. To determine a suitable dose for L002-formulated mRNA, BALB/c mice were immunized intramuscularly with different doses of LNP(L002)-mRNA DVS/MTE and LNP(SM102)-mRNA DVS/MTE, where each mRNA was 0.01 µg, 0.1µg, or 1 µg. Sera and spleen were collected at D35 (**Figure 3A**). Compared with LNP(SM102)-mRNA DVS/MTE mice, LNP(L002)-mRNA DVS/MTE mice exhibited higher neutralizing antibodies using the Delta pseudovirus. The logarithm of IC_50_ of neutralizing antibody was 1.62 vs. 2.99, 2.05 vs. 3.12, and 3.26 vs. 3.55 at 0.01 µg, 0.1 µg, and 1 µg of mRNA doses, respectively (**Figure 3B**). Notably, the logarithm of IC_50_ of neutralizing antibody at 0.01 µg mRNA with LNP(L002) was similar to 1 µg of LNP(L002)-mRNA and LNP(SM102)-mRNA (**Figure 3B**). The numbers of SFC/10^6^ splenocytes in LNP(SM102)-mRNA DVS/MTE and LNP(L002)-mRNA DVS/MTE were 2 versus 269 at 0.01 µg administration groups, 20 versus 260 at 0.1 µg administration groups and 362 versus 272 at 1 µg administration groups (**Figure 3C**). Similar T cell responses were induced at 0.01 µg dose and 1 µg dose of LNP(L002)-mRNA DVS/MTE (**Figure 3C**). Thus, L002-based LNP-mRNA formulation induced stronger T and antibody responses than SM102-based LNP mRNA formulation. Therefore, we used a low dose (0.1 µg) of LNP(L002)-mRNA in the subsequent virus challenge studies.

### LNP(L002)-formulated MTE mRNA vaccines protected mice from lethal infection of SARS-CoV-2 virus

To develop a universal COVID-19 vaccine, we propose the utilization of use two mRNAs, one encoding OVS and the other one encoding MTE. To determine if the MTE mRNA alone, and MTE plus OVS mRNAs, provide protection from SARS-CoV-2, two mouse models were used in this study, K18-hACE2 transgenic mice with SARS-Cov-2 Delta variant and BALB/c mice with mouse adapted SARS-CoV-2 MA30.

**Figure 4:**
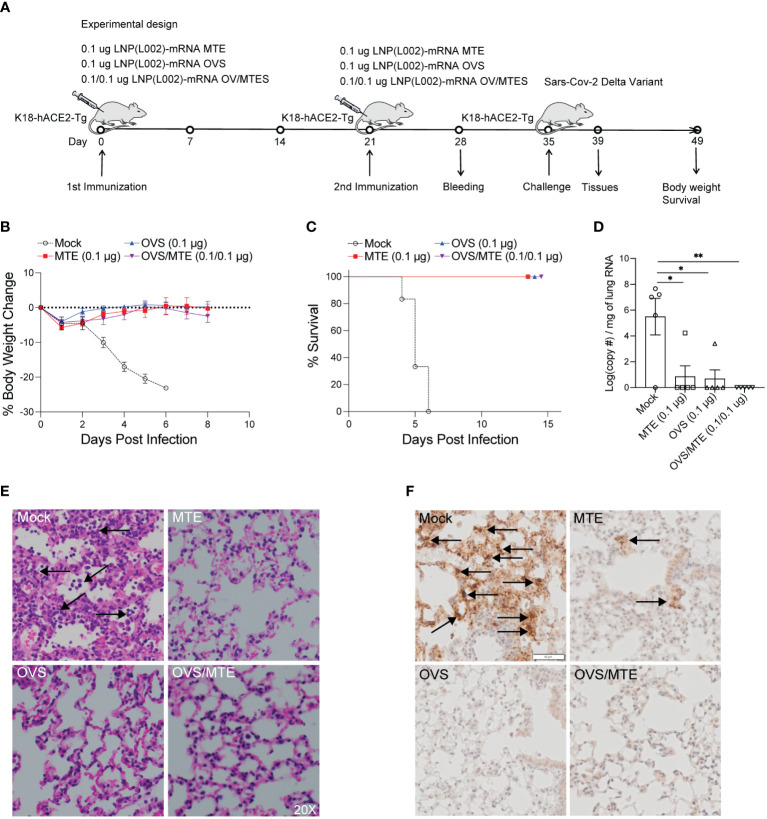
L002 formulated vaccines included LNP-mRNA OVS, LNP-mRNA MTE and LNP-mRNA OVS/MTE protected mice from SARS-CoV-2 Delta variant at a low dose. **(A)** K18-hACE2-Tg mice were immunized intramuscularly with a low dose (0.1 µg) of LNP(L002)-mRNA OVS, LNP(L002)-mRNA MTE, and LNP(L002)-mRNA OVS/MTE vaccines on D0 and D21 and challenged with a lethal dose of SARS-CoV-2 Delta variant on D35. Mouse body weight and survival were recorded after the challenge. Mouse tissues were collected 4 days after the challenge. **(B)** Mouse body weight after challenge. **(C)** Mouse survival curve after challenge. **(D)** Viral copy numbers were quantified in mouse lung tissue by qPCR. **(E)** Mouse lung was fixed and stained with H & E at 4 days after challenge. Neutrophil infiltration was marked with arrow. **(F)** Mouse lung was fixed and stained with Nucleocapsid antibody at 4 days after challenge. Arrow showed Viral particle. *P<0.05, **P<0.01.

In the first model, K18-hACE2 transgenic mice were immunized intramuscularly with 0.1 µg of LNP(L002)-mRNA OVS/MTE, LNP(L002)-mRNA MTE, LNP(L002)-mRNA OVS, or PBS at D0 and D21 and challenged with a lethal dose of SARS-CoV-2 Delta variant at D35. The body weight and survival were recorded after the challenge. Tissues were collected at four days post infection (DPI) (**Figure 4A**). We observed a significant body weight loss in the PBS group (20% reduction at six DPI), but only a slight decrease at one DPI (5%) and recovery at four DPI in all the vaccinated groups (MTE, OVS, and OVS/MTE) (**Figure 4B**). At six DPI, all of the mice in the mock group but none in the vaccinated groups died (**Figure 4C**). We also used qPCR to quantify the SARS-CoV-2 Delta variant virus in 1 mg of lung mRNA. High copies of the SARS-CoV-2 viral RNA were detected in the PBS group (1.4x10^7^), the viral RNA was below detection in the lungs from OVS/MTE group, and very low copies of the viral RNA were detected in the MTE group (3386) and OVS group (507) (**Figure 4D**). Lung histology also showed extensive neutrophil infiltration in the lungs of the PBS group but not of the MTE, OVS or OVS/MTE groups (**Figure 4E**). Similarly, a large number of viral particles were detected in the lungs of PBS group but few in MTE group and no particle in OVS or OVS/MTE group (**Figure 4F**).

**Figure 5:**
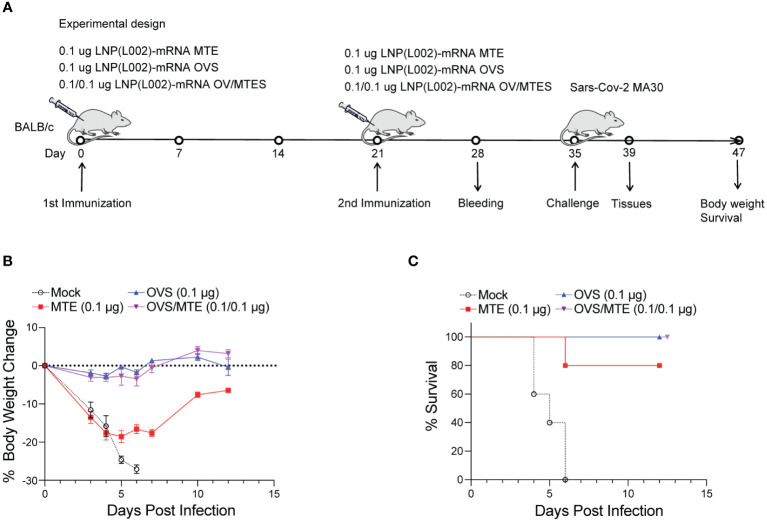
LNP(L002)-mRNA vaccines protected mice from mouse adapted SARS-CoV-2 MA30 at a low dose. **(A)** BALB/c mice were immunized intramuscularly with a low dose (0.1 ug) of LNP(L002)-mRNA OVS, LNP(L002)-mRNA MTE, and LNP(L002)-mRNA OVS/MTE vaccines on D0 and D21 and challenged with a lethal dose of SARS-CoV-2 MA30 on D35. Mouse body weight and survival were recorded after the challenge. Mouse tissues were collected 4 days after the challenge. **(B)** Mouse body weight after challenge. **(C)** Mouse survival curve after challenge.

In the second model, BALB/C mice were immunized intramuscularly with 0.1 µg of LNP(L002) formulated MTE, OVS, OVS/MTE or PBS at D0 and D21 and challenged with a lethal dose of mouse-adapted SARS-CoV-2 MA30 at D35. After challenge, mouse body weight and survival were recorded (**Figure 5A**). Similarly, mouse body weights were dramatically reduced in the PBS group (27% loss at six DPI). Although mice in MTE group also lost significant weight (19%) at five DPI but all mice recovered at twelve DPI (5% reduction). There was only a slight decrease in body weight (3%) at four DPI and recovered fully by ten DPI in the OVS and OVS/MTE groups (**Figure 5B**). At six DPI, all mice in PBS group and 1 mouse in MTE group died but none of OVS and OVS/MTE groups died (**Figure 5C**). MTE protection was weaker in this model (BALB/c background) compared to the first model (C57BL6/J), possibly due to differences in mouse strains and viruses used.

Together, these data clearly demonstrate that MTE or OVS alone can protect mice from lethal dose of SARS-CoV-2 but OVS/MTE provide the best protection.

## Discussion

Using Spike as the only vaccine target has the disadvantage that as the virus evolves, the antibodies to any given variant have decreased neutralizing activity against newly emerging variants (28–31). Beginning in September 2020, SARS-CoV-2 has rapidly created multiple VOCs (Variants of Concern), mutating from the Alpha variant to the Delta variant, then to the Omicron variant, which gave rise to additional strains, most recently BA.4 and BA.5. As these variants emerged, the efficacy of the original vaccines decreased drastically (32, 33). A bivalent booster vaccine containing Spike mRNA from the 2019 Wuhan and Omicron strains was developed by Pfizer and Moderna (34). However, as the virus mutates, these bivalent vaccines will likely become ineffective again.

Other than neutralizing antibodies, the T cell epitopes are more conserved among the viral variants (35). Therefore, T cell epitopes could provide an immense advantage to the next generation of SARS-CoV-2 mRNA vaccines. It was found that T cell numbers are reduced in multiple immune tissues and organs of COVID-19 patients, and the magnitude of the cytotoxic T lymphocytes (CTL) responses negatively correlated with the severity of the COVID-19 disease, suggesting that T cells are important for the recognition and clearance of infected cells (36). In addition, some studies have shown that virus-specific T cells can be maintained for 6 years after SARS-CoV infection (37), and are sufficient to prevent reinfection (38, 39).

MERS-CoV, SARS-CoV, and SARS-CoV-2 are coronaviruses that affect humans and may cause fatal infections (40). We compared the sequences of each epitope in MTE with MERS-CoV, SARS-CoV, SARS-CoV-2 and SARS-CoV-2 variants. T cell epitopes in MTE were 88% identical with SARS-CoV and over 94% identical with SARS-CoV-2 variants, while only 36% identical with MERS-CoV. These data showed that the sequence of MTE was highly conserved in SARS-CoV and SARS-CoV-2 variants. Although these three coronaviruses cause similar diseases and symptoms clinically, their genomic homology differs. SARS-CoV-2 have 80% genomic identity with SARS-CoV while it has only 50% identity with MERS-CoV (41). Despite the differences, we still found 24 epitopes in MTE with more than 70% similarity with MERS-CoV, which might confer some protection against MERS-CoV.

T cell immune response includes virus-specific CD8^+^ and CD4^+^ T cells, both of which play an important role in host defense against SARS-CoV-2 (42). Both the SARS-CoV-2 inactivated vaccine and the Spike mRNA COVID-19 vaccine are approved for the prevention of SARS-CoV-2 infection, and both induce comparable T cell responses. The Spike mRNA vaccine induces T cells targeting only the spike protein, whereas the inactivated vaccine targets not only the spike protein but also other viral proteins such as membrane and nucleoprotein, which are not often mutated like Spike protein. However, recently study has shown that, unlike mRNA vaccines, inactivated virus vaccines do not induce cytotoxic CD8^+^ T cells as mRNA vaccines do (43). Therefore, in the present study, we designed an MTE mRNA encoding numerous T cell epitopes from the conserved region other than Spike to elicit a broad T cell response. In addition, one study showed that an mRNA vaccine encoding nucleocapsid (N) protein alone could confer protection against SARS-CoV-2 independent of neutralizing antibodies (44). However, this study used wildtype mice, which are not naturally susceptible to SARS-CoV-2 infection, and hamsters, which do not die from SARS-CoV-2 infection (44). In addition, N protein-based vaccine elicited a strong N-specific antibody response, which may contribute to virus control by other functions of antibody other than neutralizing the virus directly. To avoid these confounding issues, we used two models in our study, the hACE2-transgenic mouse model and mouse adapted SARS-CoV-2 in wild-type mice (45). Both are commonly used to assess the vaccine efficacy against SARS-CoV-2 infection as both models mimic human infection and cause dose-dependent respiratory symptoms and lethality. In our study, the MTE mRNA encodes over one hundred T cell epitopes from SARS-CoV-2 conserved regions, including 60 MHC I epitopes and over 40 MHC II epitopes (**Supplementary Table 1**). Our results show that this MTE vaccine activates CD4^+^ T and CD8^+^ T cell responses and protects the mice from lethal infection by SARS-CoV-2 and its variants. Thus, our result unequivocally demonstrates that vaccines based on multiple T-cell epitopes can protect mice from lethal challenge and perhaps severe disease in human by activating virus-specific T-cell responses.

Although T cells cannot prevent SARS-CoV-2 from entering host cells, our studies clearly show that a T cell-inducing vaccine, in the absence of pre-existing neutralizing antibodies, can offer adequate protection against a lethal dose of SARS-CoV-2 in two mouse models. This data provides strong support for the development of T cell-vaccines as a strategy to overcome the loss of efficacy against viral variants by antibody-based vaccines. We also found in this study, that a synergistic effect was achieved if the RNA vaccine induced both neutralizing antibody and T-cell responses, this result has an important implication for vaccine design not only for COVID-19, but also for other viral infections.

Another significant observation in our study is that, compare to lipid SM102 formulated vaccines, lipid L002 formulated vaccines elicits strong T cell and neutralizing antibody response at a low dose (0.1 µg). Our results show 0.1 µg LNP(L002)-mRNA OVS is sufficient to protect mice against SARS-CoV-2. A study evaluating various dose of mRNA-1273 vaccine in mice showed comparable Spike-specific binding antibodies and protection against SARS-CoV-2 induced by 1 µg dose but not 0.1 µg dose (46). It is reported that a high dose of LNP-mRNA-based SARS-CoV-2 vaccines in mice trigger inflammatory response and result in the observed side effects (47). A benefit from low dose administration of vaccine may minimize side effects.

In conclusion, our study shows that T cell-inducing vaccines may be an effective complement to antibody-inducing vaccines and that this strategy could be applied to universal vaccine development for SARS-CoV-2 as well as other viruses.

## Data availability statement

The original contributions presented in the study are included in the article/**Supplementary Material**. Further inquiries can be directed to the corresponding authors.

## Ethics statement

Individual animal studies were reviewed and approved by Nobel Life Sciences, Bioqual Inc. and Animal Facility of Thomas Jefferson University, respectively.

## Author contributions

JQ and JJ helped conceive, design, perform, and interpret experiments. JX, LL, RM, SM, HJ and TZ provided help to JQ and JJ performed experiments. LL edited the manuscript. BM and CR-M provided technical help. LS provided SARS-CoV-2 MA30 virus, expertise, advice, and edited the manuscript. HZ and RX conceived, designed, and interpreted experiments, wrote the manuscript. All authors contributed to the article and approved the submitted version.
